# The effect of Maintenance Treatment with Twice-daily or Prolonged Once-daily Tacrolimus Formulation on Visual Evoked Potentials in Stable Kidney Transplant Recipients

**DOI:** 10.3390/jcm9061827

**Published:** 2020-06-11

**Authors:** Aureliusz Kolonko, Małgorzata Jurys, Sebastian Sirek, Tomasz Dwulit, Dorota Pojda-Wilczek, Andrzej Więcek

**Affiliations:** 1 Department of Nephrology, Transplantation and Internal Medicine; Medical University of Silesia, Francuska 20, 40-027 Katowice, Poland; tomek.dwulit@gmail.com (T.D.); awiecek@sum.edu.pl (A.W.); 2 Department of Ophthalmology, School of Medicine in Katowice, Medical University of Silesia in Katowice, Ceglana 35, 40-514 Katowice, Poland; jurysm@wp.pl (M.J.); sebasir27@wp.pl (S.S.); pojda-wilczek@wp.pl (D.P.-W.); 3 Prof. Kornel Gibiński University Clinic Centre of Medical University of Silesia, Ceglana 35, 40-514 Katowice, Poland

**Keywords:** drug interaction, kidney transplantation, optic nerve function, toxicity, visual pathway

## Abstract

In kidney transplant recipients (KTRs), uraemia-induced central nervous system damage partly subsides, while the long-lasting exposure to tacrolimus may cause pathologic visual evoked potentials (VEP) findings, which have not been investigated yet. Thus, the aim of the present study was to assess the effect of tacrolimus maintenance treatment on bioelectrical function of optic nerves in stable KTRs. Sixty-five stable KTRs were enrolled, including 30 patients treated with twice-daily (Prograf) and 35 patients treated with prolonged once-daily (Advagraf) tacrolimus formulation. In all patients, pattern and flash VEP measurements were performed. Tacrolimus dosing and exposure were also analyzed. Overall, 129 eyes were analyzed. In pattern VEP, both (1°) and (15′) latencies of P100 waves were significantly longer, whereas (1°) and (15′) amplitudes were lower in the Advagraf group as compared with the Prograf group. Multivariate regression analyses revealed that the use of Advagraf (vs. Prograf) was independently associated with longer (1°) and (15′) P100 latencies and lower corresponding amplitudes, whereas log tacrolimus daily dose was only related to amplitudes in a whole study group. In flash VEP, log tacrolimus trough level was associated with negative changes in P2 wave amplitude irrespective of tacrolimus formulation, whereas its association with P2 latency was observed only in the Prograf group. Both the type of tacrolimus formulation and drug exposure influenced the VEP parameters in stable KTRs.

## 1. Introduction

Nowadays, tacrolimus is the main immunosuppressive agent used in kidney transplantation [1]. The introduction of this potent calcineurin inhibitor into the clinical practice improved early and long-term kidney graft outcomes, mostly as a consequence of the reduction of acute rejection episodes [2]. However, other than the variety of adverse effects, namely post-transplant diabetes mellitus, nephrotoxicity, electrolyte disturbances and increased rate of infectious complications, tacrolimus can also induce neurotoxicity [3]. Mild neurological complications include headache, paresthesia, tremor, sleep disturbances and photophobia, while more severe symptoms, such as confusion, seizures, dysarthria, vision loss and encephalopathy may also occur [3,4,5]. Nonetheless, their mechanisms are not fully elucidated, being perhaps intermediated by oligodendrocytes, glia and vascular endothelium injury [6]. All the above-described symptomatology may be seen both in the early post-transplant period and in stable kidney transplant recipients (KTRs) [5,7]. Moreover, the tacrolimus-induced peripheral nerve dysfunction may proceed without a clinical manifestation, even if tacrolimus blood levels are within the therapeutic range. Thus, there is a need for a diagnostic procedure, which might be useful in the early detection of tacrolimus-induced neurotoxicity.

Visual-evoked potentials (VEP) is a recorded occipital lobe brain wave potential in response to visual stimulation [8]. It has been demonstrated to provide objective information on the functional integrity of central nervous system structures, being the most useful in diagnosis of demyelinating, brainstem or sensory organ disease [9]. Out of all electrophysiological examinations, pattern reversal VEP (PVEP) spectrum abnormalities are the most early and frequently observed in uremic patients [10], with delayed latency and reduced amplitude of the P100 component as typical findings [9,10]. In a substantial proportion of uremic patients, these anomalies are detected without any pathologies in neurological examination [11]. After a successful kidney transplantation, the uremia-induced metabolic disturbances subside, however, the lack of complete normalization of VEP recordings may be explained by the optic nerve dysfunction caused by calcineurin inhibitor treatment [9,10,12,13]. The presence of tacrolimus-associated neurotoxicity in liver transplant patients without previous uremia confirm such a hypothesis [14,15]. To date, any studies reporting the association between tacrolimus exposure and VEP results in KTRs are lacking. Of interest, when the once-daily, prolonged release tacrolimus formulation was introduced to the market, both an improvement in drug adherence and a better safety profile was anticipated based on substantial pharmacokinetic differences, which included lower peak levels, but similar drug exposure as compared to the twice-daily original preparation [16]. Indeed, although potentially beneficial metabolic changes were observed in patients using once-daily tacrolimus formulation [17,18], to date there is virtually no data comparing the neurotoxicity of both drug formulations. Thus, the aim of the present study was to assess the effect of maintenance treatments with two different formulations of tacrolimus on the bioelectrical function of the optic nerve in stable KTRs.

## 2. Material and Methods

### 2.1. Study Group

Sixty-five adult recipients of their first kidney graft, who had undergone a successful kidney transplantation at least 24 months previously, were enrolled to this cross-sectional study. The study was conducted in accordance with the Declaration of Helsinki. The Bioethics Committee of the Medical University of Silesia granted permission for the study protocol (No KNW/0022/KB1/93/13) and informed consent was obtained from all patients.

At the start, based on the prospectively operated center database, we identified those patients who were treated with tacrolimus since transplantation. Patients with pre-transplant diabetes and those with diabetes diagnosed at any time post-transplantation were excluded. During the routine ambulatory visit, patients were examined by an ophthalmologist. At this stage, we further excluded patients with photosensitive epilepsy; a history of strokes; neurosurgery episodes; serious head injury; a history of optic nerve and/or retina diseases; cataract worsening visual acuity; nystagmus; photophobia or other unpleasant sensations due to flashes of light; and concomitant therapy with potentially neurotoxic medications other than tacrolimus. Furthermore, we excluded patients with unstable kidney graft function over the last 6 months, current infections, a diagnosis of malignancy or liver cirrhosis.

In all study patients, intima-media thickness (IMT) was measured and the occurrence of any plaque in both carotid arteries were noted as surrogate markers of atherosclerosis. Additional blood samples were withdrawn for the several biochemical parameters determination and for the calculation of tacrolimus exposure using the area under the curve (AUC). Both pattern and flash VEP measurements were also performed.

### 2.2. Carotid Sonography

Carotid ultrasound was performed using a Siemens machine (Sonoline Antares, Mountain View, CA, USA), equipped with a 4.0–9.0 MHz linear transducer. Carotid arteries were examined with a patient in the supine position with the neck extended. The evaluation included the common, internal, and external carotid arteries, and a carotid bifurcation on each side. The common carotid artery IMT was measured within 2 cm proximal to the carotid bulb, omitting any visible plaques, and the higher value from both sides was taken for further analyses. The results from three separate measurements on each side were averaged. At each examined location, the vessels were carefully evaluated in terms of the presence of plaque, which was classified based on the simplified scale: 0—no lesions; 1—non-calcified lesions; 2—at least one calcified lesion; 3—few calcified lesions; 4—carotid bulb heavily covered by calcified lesions.

### 2.3. Laboratory Parameters

The plasma concentrations of high-sensitivity C-reactive protein (hsCRP) were assessed with the use of an enzyme-linked immunosorbent assay (ELISA) (Immundiagnostic AG, Bensheim, Germany), with the limit of quantification (LoQ) of 0.09 mg/l, intra-assay variation <6%, inter-assay variation <11.6%. Plasma concentrations of interleukin-6 (IL-6), tumor necrosis factor alpha (TNF-α) and brain derived neurotrophic factor (BDNF) were assessed with an ELISA (R&D Systems, Minneapolis, Minnesota) with an LoQ of 0.7 pg/mL, 0.191 pg/mL and 20 pg/mL, intra-assay variation <4.2%, <8.7% and <6.3%, and inter-assay variation <6.4%, <10.4%, and <11.4% respectively. Plasma concentrations of asymmetric dimethylarginine (ADMA) were measured using ELISA (Immundiagnostik, AG, Bensheim, Germany), with an LoQ of 0.16 µmol/l, intra-assay variation <7.6% and inter-assay variation <4%. Plasma concentrations of endothelin-1 (ET-1) were measured using ELISA (USCN Life Sciences, Wuhan, China), with an LoQ of 2.71 pg/mL, an intra-assay variation <10%, inter-assay variation <12%.

Kidney graft function was measured using an estimated glomerular filtration rate (eGFR) calculated according to the Modification of Diet in Renal Disease (MDRD) formula.

### 2.4. Tacrolimus Exposure Measurements

Blood samples for tacrolimus trough level assessments were routinely withdrawn 12 h after the evening dose in patients receiving twice-daily formulation (Prograf) and 24 h after the morning dose in patients treated with once-daily, prolonged-release formulation (Advagraf), immediately prior to the next morning dose. Additional blood samples for AUC calculation were withdrawn 3 h after the morning tacrolimus dose ingestion. Tacrolimus concentrations were assessed using the microparticle enzyme immunoassay for Abbott Architect (MEIA; Abbott Laboratories, Abbott Park, IL, USA). AUC for tacrolimus was calculated using the formula:

AUC = 13.17 + (5.43 × “Tc 0”) + (4.72 × “Tc 3”)(1)

where Tc 0 means tacrolimus blood level before the morning dose (trough level) and Tc 3 means tacrolimus blood level measured 3 h after the morning dose ingestion [19]. The time-weighted tacrolimus exposure during the whole year preceding the time of study examination was calculated based on all consecutive tacrolimus blood trough levels measured during the last 12 months prior to the study time-point. The formula was as follow:

time-weighted tacrolimus exposure = (1/2 × (1Tc level + 2Tc level) × time between 1Tc and 2Tc levels) + (1/2 × (2Tc level + 3Tc level) × time between 2Tc and 3Tc levels) + ….(2)

where 1Tc, 2Tc etc. means the consecutive tacrolimus blood trough levels measured during the whole year.

### 2.5. Visual Evoked Potential Measurement

In all eligible patients, VEP were tested using the Reti-Port electrophysiological apparatus from Roland Consult (Brandenburg a.d.Havel, Germany), in accordance with the standards of the International Society for Clinical Electrophysiology of Vision (ISCEV) [20]. The patient’s scalp was glued with gold-cup electrodes using abrasive paste (Nuprep Skin Prep Gel, Weaver and Company, Aruroa, CO, USA) and conductive paste (Ten20 Conductive Paste, Weaver and Company, Aruroa, CO, USA), according to the international system 10/20: active electrodes located at the Oz point (above the occipital area) in relation to the electrode at the Fz point (above the frontal area). The ground electrode was glued at point Cz (above the parietal area).

A black and white checkerboard pattern with alternating phase change (pattern reversal VEP, PVEP) was used to elicit a visual cortical response. The reversal rate was of 2 reversals per second. Each patient’s eye was stimulated with small (0.25°, i.e., 15′ per arc) and large (1° per arc) checks. For flash stimulation (flash VEP, FVEP), standard flashes with a frequency of 1.4 Hz were used in the Ganzfeld stimulator. Each VEP test was preceded by checking the visual acuity of the distance in the patient’s correction with glasses using Snellen charts, an intraocular pressure test using a Goldmann applanation tonometer (CSO, Firenze, Italy) and was finished with anterior and posterior examination of the eyeball in a slit lamp (Haag-Streit) with an indirect method using a Volka lens (Super Field) to monitor possible pathological changes in the eye that may affect the result of the VEP test or the result of neurotoxicity (primarily changes in the optic disc: pallor, blurring or raising boundaries, etc.). Before the fundus examination, short-acting (4–6 h) mydriatics were given: parasympatholytic (1% Tropicamidum, Polfa, Poland) or sympathomimetic (10% Neosynephrin-POS, Ursapharm, Poland) drops. In case of a lack of patient consent for mydriasis, the fundus was checked through the narrow pupil. Refusal of consent for pupil dilation did not affect the patient’s further participation in the study and did not change the patient’s treatment.

The amplitude and the latency of the P100 (PVEP) and P2 (FVEP) waves were analyzed. Amplitudes were measured from the top of the negative peak (N) preceding the positive (P) wave (N75-P100 and N2-P2) and the latency from the beginning of the stimulation to the occurrence of the highest value of the marked wave.

### 2.6. Statistical Analysis

Statistical analyses were performed using the STATISTICA 13.3 PL for Windows software package (Tibco Inc., Palo Alto, CA, USA) and MedCalc v19.2.1 (MedCalc Software, Mariakerke, Belgium). Values are presented as means and 95% confidence intervals or frequencies. Variables with skewed distribution were presented as medians and interquartile ranges (IQRs). The main comparison was performed for groups with different tacrolimus formulations: Prograf or Advagraf. The subgroups were compared using the Student t test or χ^2^ test (for variables with normal distribution) or the Mann–Whitney U test (for variables with a not-normal distribution). Correlation coefficients were calculated according to Pearson. Data of variables with a not-normal distribution were logarithmized for the correlation analyses. Multivariate regression analyses were performed for the (1°) and (15′) latencies of the P100 wave as dependent variables, including age, log time post transplantation, 12-month tacrolimus exposure, log tacrolimus trough level and the tacrolimus formulation (Advagraf vs. Prograf) as potential independent variables. Analogic regression analyses were performed for the (1°) and (15′) amplitudes of the P100 wave as dependent variables, including eGFR, log time post transplantation, 12-month tacrolimus exposure, log tacrolimus daily dose and the tacrolimus formulation (Advagraf vs. Prograf) as potential independent variables. Lastly, we also performed the backward multivariate regression analysis for P2 wave latency (including log tacrolimus trough level, log tacrolimus dose per kg, body mass index (BMI), 12-month tacrolimus exposure, age and the tacrolimus formulation as potential independent variables) and P2 wave amplitude (including log tacrolimus trough level, log tacrolimus dose per kg and the tacrolimus formulation as potential independent variables). In all statistical tests, *p* values less than 0.05 were considered as statistically significant.

## 3. Results

### 3.1. Study Group

All study patients were treated with tacrolimus since the transplantation. Thirty patients received a twice-daily formulation (Prograf) and 35 study participants received a once-daily tacrolimus formulation (Advagraf) at least during the 12 months preceding the study measurements (after conversion from the initial Prograf treatment). The additional immunosuppressive regimen consists of mycophenolate mofetil or sodium and steroids. The mycophenolate daily doses and the percentage of patients receiving steroids at the time of the study were comparable between subgroups.

Both study subgroups did not differ in terms of age, sex, BMI, dialysis vintage prior to transplantation and kidney graft function at the time of the study. Both subgroups were also comparable as regard to the maximum IMT value and the percentage of calcified plaques in carotid arteries. However, the post-transplant follow-up period was significantly longer in the Advagraf-treated patients (Table 1). Importantly, there were no significant differences in plasma concentrations of inflammatory markers (hsCRP, IL-6 and TNF-α) between the study subgroups.

### 3.2. Tacrolimus Exposure

At the time of the study, there were no differences between the daily tacrolimus dose and the tacrolimus dose calculated per kg of body weight between the study subgroups (Table 1). In contrast, the tacrolimus blood trough level was significantly higher in patients treated with Prograf (6.3 vs. 5.8 ng/mL) as compared with patients receiving Advagraf. In line with this, the calculated 12-month overall tacrolimus exposure based on the consecutive pre-dose troughs, was also higher in patients receiving Prograf (Table 1), whereas the tacrolimus AUC at the time of the study and an estimated 12-month exposure calculated per kg of body weight were comparable between the two subgroups.

### 3.3. Visual Evoked Potentials (VEP)

**Figure 1:**
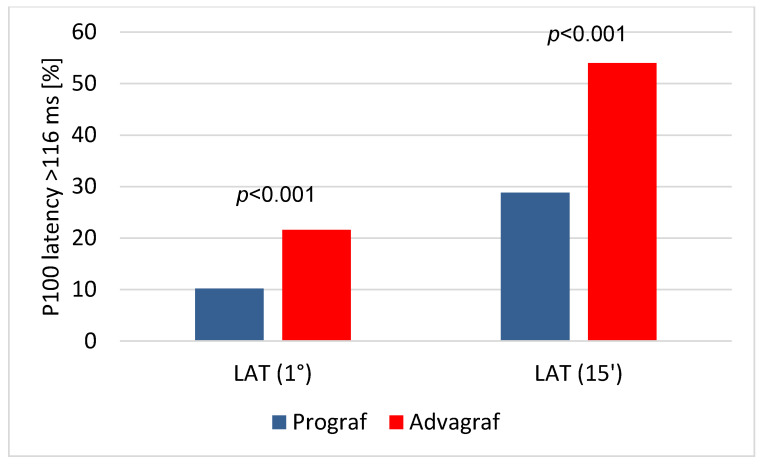
Results of the pattern visual evoked potentials (VEP) examination. The percentage of eyes with prolonged P100 latency (1°) and latency (15′) duration (with laboratory normal value ≤116 ms) in patients treated with Prograf (blue bars) and Advagraf (red bars).

In the pattern VEP examinations, both (1°) and (15′) latencies were significantly longer in the Advagraf group as compared with the Prograf group (Table 2). Accordingly, the percentage of eyes with both latency (1°) and latency (15′) (Figure 1) duration >116 ms were significantly higher in the Advagraf group. Moreover, the (1°) and (15′) amplitudes were significantly lower in the Advagraf group as compared to the Prograf group, although there were no differences in the percentage of eyes with amplitude <10 µV (Table 2). P100 wave (1°) latency positively correlated with (15′) latency (r = 0.290; *p* < 0.001) and inversely with (1°) and (15′) amplitudes (r = −0.201; *p* < 0.05 and r = −0.243; *p* < 0.001, respectively). Except for the above associations, only (1°) and (15′) amplitudes were positively related (r = 0.767; *p* < 0.001).

In the flash VEP examinations, there were no significant differences between the groups with regard to the latency and amplitude of the P2 wave. P2 wave latency weakly correlated only with (15′) P100 latency (r = 0.222; *p* < 0.05), whereas log P2 wave amplitude was positively associated with both (1°) and (15′) P100 amplitudes (r = 0.361 and r = 0.327) and inversely with P2 latency (r = −0.327); all *p* < 0.001.

### 3.4. Association of VEP Parameters, Clinical Characteristics and Tacrolimus Exposure

**Figure 2:**
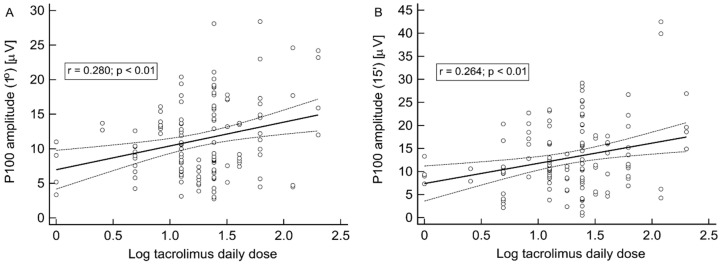
Results of the pattern VEP examination. The correlation between log tacrolimus daily dose and P100 wave (1°) (**A**) or (15′) (**B**) P100 amplitudes.

In univariate analyses, P100 wave (1°) and (15′) latencies (r = 0.342 and r = 0.389, respectively; both *p* < 0.001), but not amplitudes were significantly associated with recipient age. On the other hand, P100 wave (1°) and (15′) amplitudes, but not corresponding latencies, were significantly positively associated with log tacrolimus daily dose (r = 0.280 and r = 0.264, respectively; both *p* < 0.01) (Figure 2A and 2B) and log tacrolimus dose per kg of body weight (r = 0.341 and r = 0.299, respectively, both *p* < 0.001), whereas both were inversely related to eGFR (r = −0.233; *p* < 0.01 and r = −0.394; *p* < 0.001, respectively). Notably, there were no associations between P100 wave (1°) and (15′) latencies and amplitudes and log tacrolimus trough concentration and tacrolimus AUC.

**Figure 3:**
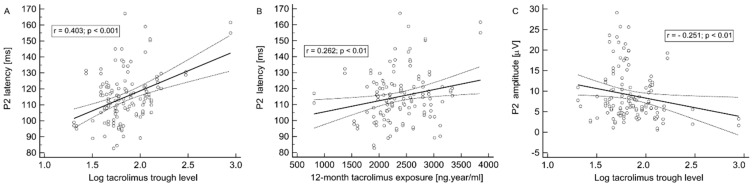
Results of the flash VEP examination. The associations of P2 wave latency and log tacrolimus trough level (**A**) and 12-month tacrolimus exposure (**B**). The association of P2 wave amplitude and log tacrolimus trough level (**C**).

In contrast, P2 wave latency presents its most pronounced associations with log tacrolimus trough level (r = 0.403; *p* < 0.001) (Figure 3A), log tacrolimus dose per kg of body weight (r = −0.177; *p* < 0.05), BMI (r = 0.311; *p* < 0.001), 12-month tacrolimus exposure (r = 0.262; *p* < 0.01) (Figure 3B) and age (r = 0.221; *p* < 0.05), but not with tacrolimus daily dose or eGFR. On the other hand, P2 wave amplitude inversely correlated with log tacrolimus trough level (r = −0.251; *p* < 0.01) (Figure 3C) and log tacrolimus dose per kg of body weight (r = 0.232; *p* < 0.01), but not with log tacrolimus daily dose, 12-month tacrolimus exposure or eGFR.

**Figure 4:**
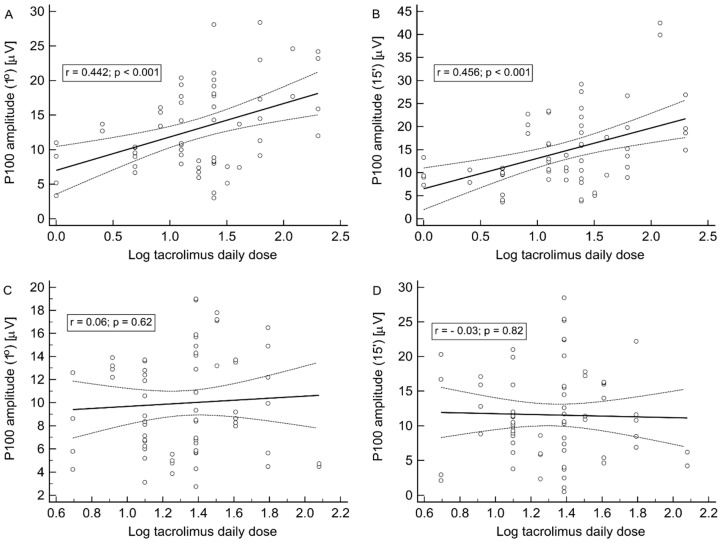
The pattern VEP examination. The associations of both P100 wave (1°) and (15′) amplitudes with log tacrolimus daily dose in the Prograf (**A**) and (**B**) and the Advagraf (**C**) and (**D**) subgroups.

Of interest, the type of tacrolimus formulation implicated noteworthy differences concerning some associations described above in the whole study group. Namely, the strong positive correlations of both P100 wave (1°) and (15′) amplitudes with log tacrolimus daily dose (r = 0.442 and r = 0.456, respectively; both *p* < 0.001) (Figure 4A,B) and log tacrolimus dose per kg of body weight (r = 0.561 and r = 0.566, respectively; both *p* < 0.001) were observed only in the Prograf subgroup, whereas no analogical associations were observed in the Advagraf subgroup (Figure 4C,D).

Similarly, all aforementioned associations between P2 wave latency and other parameters were observed in the Prograf (r = 0.564; *p* < 0.001, r = 0.254; *p* < 0.05, r = 0.307; *p* < 0.01 and r = 0.281; *p* < 0.05, respectively), but not the Advagraf, subgroups, except the correlation with BMI, which was universal. Out of the previously described P2 wave amplitude correlations, only the one with tacrolimus trough level was universal, although it was of borderline significance in both subgroups, whereas others were present only in the Prograf subgroup.

### 3.5. Univariate Analyses of VEP Parameters and Biochemical Vascular Markers

In the whole study group, ET-1 concentration was positively associated with P100 wave (1°) and (15′) amplitudes (r = 0.202; *p* < 0.05 and r = 0.258; *p* < 0.01, respectively), but not corresponding latencies. Interestingly, plasma ADMA and BDNF concentrations were not associated with P100 (1°) and (15′) measures in the whole group, but both were positively correlated with P100 (15′) latency only in the Advagraf subgroup (r = 0.311 and r = 0.315; both *p* < 0.05).

### 3.6. Univariate Analyses of VEP Parameters and Inflammatory Status Markers

The analysis of inflammatory status parameters revealed that IL-6 and TNF-α, but not hsCRP concentrations showed positive associations with P100 (1°) and (15′) amplitudes (for log IL-6: r = 0.182; *p* < 0.05 and r = 0.297; *p* < 0.01, respectively; for log TNF-α: r = 0.210; *p* < 0.05 and r = 0.329; *p* < 0.001, respectively). There was no significant association between those biochemical parameters and P100 latencies.

### 3.7. Multivariate Analyses

Stepwise backward multivariate regression analysis revealed that age (partial correlation coefficient (r_partial)_ = 0.309; *p* < 0.001) and the use of Advagraf formulation (r_partial_ = 0.262; *p* < 0.01), but not log time post transplantation or tacrolimus 12-month exposure, independently influenced the P100 wave (1°) latency (R^2^ = 0.18). In an analogic analysis, age (r_partial_ = 0.432; *p* < 0.001), log time post transplantation (r_partial_ = −0.195; *p* < 0.05) and the use of Advagraf formulation (r_partial_ = 0.178; *p* < 0.05), but not tacrolimus exposure, independently influenced the P100 wave (15′) latency (R^2^ = 0.22).

Stepwise backward multivariate regression analysis revealed that the use of Advagraf formulation (r_partial_ = −0.349; *p* < 0.001), log tacrolimus daily dose (r_partial_ = 0.294; *p* < 0.001) and eGFR (r_partial_ = −0.207; *p* < 0.05), but not log time post transplantation or tacrolimus exposure, independently influenced the P100 wave (1°) amplitude (R^2^ = 0.21). In an analogic analysis, eGFR (r_partial_ = −0.376; *p* < 0.001), log tacrolimus daily dose (r_partial_ = 0.256; *p* < 0.01) and the use of Advagraf formulation (r_partial_ = −0.272; *p* < 0.01) independently influenced the P100 wave (15′) latency (R^2^ = 0.25).

Stepwise backward multivariate analysis revealed that log tacrolimus blood trough level (r_partial_ = 0.319; *p* < 0.001), BMI (r_partial_ = 0.213; *p* < 0.01) and age (r_partial_ = 0.213; *p* < 0.05) independently influenced the P2 wave latency (R^2^ = 0.24). In a similar analysis, log tacrolimus dose per kg (r_partial_ = 0.217; *p* < 0.05) and log tacrolimus blood trough level (r_partial_ = −0.177; *p* < 0.05) independently influenced the P2 wave amplitude (R^2^ = 0.09).

## 4. Discussion

The main finding and the novelty of the present study is the association between tacrolimus exposure and parameters of both pattern and flash VEP spectra in stable non-diabetic KTRs. Moreover, the negative effect on the bioelectrical function of the visual pathway seemed to be more intensified in patients treated with once-daily tacrolimus formulation. Those independent relationships were confirmed in multivariate regression analyses.

The rationale for the use of VEP measurements as a diagnostic tool in a clinical setting of tacrolimus neurotoxicity in the kidney transplant population is based on several premises. The animal models support the concept that the VEP provides a highly sensitive measure to detect demyelination in optic neuritis [21]. However, although the VEP may detect optic nerve dysfunction, it is not helpful in determining causes [8]. VEP response reflects the pathway from the retina to area 17 in the occipital cortex, so a normal P100 spectrum does not exclude lesions beyond area 17. Interestingly, in a case of tacrolimus-associated optic neuropathy in a pancreatic islet transplant recipient, PVEP showed a delay in P100 latency and a concomitant decrease in amplitude. One month after tacrolimus withdrawal, along the improvement of visual acuity, PVEP results improved [12]. A unilateral VEP abnormality suggests an optic neuropathy if ocular diseases can be ruled out [8]. Thus, to increase the precision of our present investigation, we excluded any current or past clinical evidence, including diabetes, which could potentially have interfered and modified the VEP results.

As the tacrolimus molecule has a lipophilic nature, the postulated mechanism of tacrolimus neurotoxicity may involve an altered blood–brain barrier permeability due to increased radical oxygen species and ET-1 production (5). The latter may result in vasoconstriction and relative ischemia of macular optic fibers [12]. Interestingly, the vasoconstrictive effect of tacrolimus on renal microcirculation is mediated by a nitric oxide blockade [22] and causes a higher intrarenal resistance index (RI) measured by Doppler ultrasound as compared with m-TOR inhibitor immunosuppressive regimen [23]. Moreover, in patients with unilateral optic neuritis confirmed by pathological VEP results, unilateral changes in orbital hemodynamics were found, including the asymmetrically increased RI values [24,25]. Taking into account the abovementioned evidence together with the positive association between RI values measured in renal and orbital arteries [26], we may speculate that VEP measurement might reflect the tacrolimus-induced changes in the bioelectrical function of the optic nerve.

In the present study, we have analyzed both pattern and flash VEP results. Of importance, when both latency and amplitude of the P2 component were negatively influenced by the increased blood tacrolimus trough concentration, the observed association between the tacrolimus dose and P100 wave amplitude was positive, i.e., there was a tendency toward a normal P100 spectrum along with an increasing drug dose. This may be partly explained by the unexpectedly positive correlation between ET-1 concentration and P100 amplitude values in our study, as increased ET-1 levels and vasoconstriction were previously assigned to tacrolimus nephrotoxicity [27]. Thus, we confirmed the independent association between tacrolimus dosing/blood levels and PVEP and FVEP abnormalities, but these relationship seems to be ambiguous and clinically inconclusive. We may only speculate that those discrepancies could result from the different origin of PVEP and FVEP responses.

In contrast, the use of once-daily tacrolimus formulation was independently associated with all identified unfavorable tendencies in the P100 wave spectrum in comparison with twice-daily formulation. As the Advagraf subgroup was characterized by a substantially longer observation after transplantation (i.e., the time exposure to potential neurotoxic effects), we cannot exclude the potential bias. However, the negative association between the time after transplantation and P100 (15′) latency as well as the lack of such associations with the rest of PVEP or FVEP parameters in multivariate analyses does not confirm such an explanation. It is also worth noticing that a positive correlation between P100 (15′) latency and BDNF level was seen only in the Advagraf subgroup. BDNF regulates synaptic plasticity and is a critical protein in maintaining neuronal function [28]. On the other hand, as increased BDNF expression in specific central nervous system regions and release was previously observed both in tacrolimus-treated and diabetic rats [28] and in astrocytes in vitro [29], with the additional cumulative effect [28], it may play a role in tacrolimus-induced neurotoxicity. Of note, all those experimental models were realized using twice-daily formulation. Another issue of potential importance is the discrepancy between the observed associations of VEP parameters and the different measures of patient’s exposure to tacrolimus, i.e., daily dose, trough level, estimated 12-month exposure and AUC. However, it has been shown that AUC-guided tacrolimus dosing prevents progressive systemic overexposure in KTRs [30]. Moreover, due to specific pharmacokinetic relationships among AUC, peak and trough levels, the blood trough level is the preferable measure for tacrolimus therapy monitoring [31].

Interestingly, the prolonged, once-daily formulation was developed to increase the drug adherence and minimize toxicity due to the more flattened blood level slope. In fact, beneficial changes were noted in renal hemodynamics and function after conversion to prolonged-release tacrolimus in healthy volunteers [32]. However, in the only study comparing those tacrolimus formulations performed in liver transplant recipients, previously encephalopathic subjects were at greater risk of developing early neurotoxicity in the Advagraf subgroup [33]. Moreover, all Advagraf-treated patients needed the rescue conversion to cyclosporine due to the worse clinical course and the time to such a conversion was shorter in this subgroup [33]. However, the explanation of the reported differences between both tacrolimus formulations as regard to clinical and subclinical neurotoxicity remains unknown.

Nowadays, an issue with tacrolimus neurotoxicity is emerging as the another prolonged-release tacrolimus formulation was introduced with a different mechanism of drug absorption, based on Melt-Dose technology [34]. Notably, one of main marketing strategies concerning this new tacrolimus formulation—Envarsus—is based on its suggested lower neurotoxicity, as assessed by the degree of tremor [35]. However, the precise measurement of tremor is technically difficult and the literature yields conflicting results [35,36]. Hence, VEP measurements seem to be more reliable for quantitative assessment of neurotoxicity and may be useful for further comparative studies in this field.

The main drawback of our study is the limited number of participants. This is mostly the consequence of the strict study exclusion criteria, which covered any diabetes or previous neurologic disease. Such a selection aimed to eliminate patients with comorbidities that may have interfered with the VEP results. Nevertheless, this is the first clinical study analyzing the relationship of tacrolimus dosing and exposure with the bioelectrical function of the visual pathway measured by VEP. Another limitation is the lack of a control group of KTRs receiving calcineurin inhibitor-free immunosuppressive regimen. This is because of the unavailability of such patients who met the study inclusion criteria in our out-patient department KTR cohort. Moreover, as we used a limited sampling strategy (LSS) for tacrolimus AUC calculation which was originally developed for liver transplant patients, it may not be ideal for kidney transplant recipients. However, the analysis of several LSS developed based on different solid organ recipients populations [37] indicated that implementation of LSS based on regression analysis could produce satisfactory predictions as compared with classic AUC determination. Lastly, the statistically significant differences between study subgroups (regarding the time after transplantation and differences in some measures of tacrolimus exposure) might represent a potential bias. Nevertheless, there was no difference in AUC values, which is the gold standard to assess drug exposure. Moreover, we included all abovementioned parameters in the multivariate analyses.

In conclusion, in stable KTRs treated with tacrolimus, pathologic values in VEP examination are highly prevalent despite no overt tacrolimus neurotoxicity. There is an association between tacrolimus exposure and simultaneously assessed bioelectrical function of the visual pathway in kidney transplant population. The substantial differences in VEP findings between subjects treated with twice-daily or once-daily tacrolimus formulations were also described. Further investigation is warranted to confirm these findings, including the conversion study switching the tacrolimus formulations with concomitant accurate neurotoxicity monitoring.

## Figures and Tables

**Table 1:** The characteristics of kidney transplant recipients treated with two different tacrolimus formulations.

	Study Group *n* = 65	Prograf *n* = 30	Advagraf *n* = 35	*p*
Clinical characteristics				
Age (years)	46.6 (43.9–49.4)	44.8 (40.9–48.9)	48.2 (44.3–52.2)	0.21
Gender (M/F)	38/27	17/13	21/14	0.79
BMI (kg/m^2^)	26.4 (25.3–27.5)	26.1 (24.6–27.5)	26.7 (25.0–28.3)	0.58
Dialysis vintage (months) *	24 (13–34)	24 (13–36)	23 (13–31)	0.68 **
Time post transplantation (months) *	30 (27–86)	28 (25–31)	60 (29–93)	<0.001 **
eGFR (ml/min/1.73 m^2^)	58.2 (52.8–63.5)	59.5 (52.1–67.0)	57.0 (49.0–65.0)	0.64
MMF dose (mg/day) *	1000 (750–1000)	1000 (1000–1000)	1000 (500–1000)	0.26 **
Steroid use (*n* (%))	45 (69.2)	20 (66.7)	25 (71.4)	0.68
IMT (mm)*	0.6 (0.6–0.7)	0.6 (0.6–0.7)	0.6 (0.6–0.8)	0.50 **
Calcified plaques (*n* (%))	28 (43.1)	12 (40)	16 (46)	0.64
Tacrolimus exposure				
Daily tacrolimus dose (mg) *	4.0 (3.0–4.5)	3.8 (2.5–4.5)	4.0 (3.0–4.5)	0.49 **
Tacrolimus dose/kg of body weight (mg/kg) *	0.047 (0.036–0.062)	0.047 (0.034–0.061)	0.047 (0.039–0.068)	0.44 **
Tacrolimus trough level (ng/mL) *	6.2 (5.4–7.4)	6.3 (5.8–7.8)	5.8 (5.2–7.4)	<0.01 **
Tacrolimus AUC (ng.h/mL)	104.6 (96.0–113.3)	105.8 (87.0–124.7)	104.1 (93.7–114.5)	0.44
Estimated 12-month tacrolimus exposure (ng.year/mL)	2375 (2251–2499)	2483 (2275–2692)	2282 (2133–2430)	<0.01
Estimated 12-month tacrolimus exposure calculated per kg of body weight	31.7 (29.5–33.9)	32.4 (29.3–35.5)	31.1 (28.0–34.3)	0.18
Laboratory parameters				
hsCRP (mg/l) *	2.0 (1.2–4.3)	1.9 (1.2–4.3)	2.3 (1.2–5.7)	0.22 **
IL-6 (pg/mL) *	2.1 (1.6–2.7)	2.0 (1.6–2.5)	2.2 (1.6–2.7)	0.38 **
TNF-α (pg/mL) *	1.8 (1.3–2.4)	1.6 (1.3–2.0)	1.8 (1.4–2.4)	0.11 **
ET-1 (pg/mL)	20.1 (17.9–22.3)	19.5 (16.7–22.4)	20.6 (17.2–24.0)	0.63
ADMA (µmol/l)	0.92 (0.83–1.00)	1.03 (0.89–1.17)	0.82 (0.71 -0.93)	0.02
BDNF (pg/mL) *	1760 (1120–5348)	1620 (1120–5348)	2647 (1256–4980)	0.67 **

Data presented as means with 95% Confidence Interval, except * medians with interquartile range. Statistics: Student t test or χ^2^ test, except ** U Mann–Whitney test, for comparison between the Prograf and Advagraf subgroups. BMI, body mass index; eGFR, estimated glomerular filtration rate according to MDRD formula; IMT, carotid artery intima-media thickness; AUC, area under the curve for tacrolimus; hsCRP, high sensitive C-reactive protein; IL-6, interleukin-6; TNF-α, tumor necrosis factor α; ET-1, endothelin 1; ADMA, asymmetric dimethylarginine; BDNF, brain-derived neurotrophic factor.

**Table 2:** Results of pattern and flash VEP examinations in the eyes of kidney transplant recipients treated with two different tacrolimus formulations.

	Prograf *n* = 59	Advagraf *n* = 70	Statistics
Pattern VEP examination—P100 parameters			
Latency (1°) (ms)	105.5 (104.2–106.9)	109.2 (107.4–111.2)	<0.01
Latency (1°) > 116 ms (*n* (%))	6 (10.2)	21 (27.6)	<0.001
Latency (15′) (ms)	111.7 (109.7–113.7)	114.8 (112.6–117.0)	<0.05
Latency (15′) > 116 ms (*n* (%))	17 (28.8)	41 (54.0)	<0.001
Amplitude (1°) (µV)	12.9 (11.3–14.5)	10.1 (9.0–11.1)	<0.01
Amplitude (1°) < 10 µV (*n* (%))	25 (42.4)	37 (56.6)	0.24
Amplitude (15′) (µV)	14.4 (12.2–16.5)	11.8 (10.3–13.4)	<0.05
Amplitude (15′) < 10 µV (*n* (%))	19 (32.2)	24 (39.5)	0.80
Flash VEP examination—P2 parameters			
Latency (ms)	115.4 (110.7–120.2)	116.1 (112.8–119.4)	0.82
Latency > 130 ms (*n* (%))	11 (18.6)	8 (11.4)	0.25
Amplitude (μV) *	6.3 (4.4–11.5)	7.9 (5.8–12.7)	0.24 **

Data presented as means with 95% Confidence Interval, except * medians with interquartile range. VEP, visual evoked potentials. Statistics: Student t test or χ^2^ test, except ** Mann–Whitney U test.
